# A dual sgRNA-directed CRISPR/Cas9 construct for editing the fruit-specific β-cyclase 2 gene in pigmented citrus fruits

**DOI:** 10.3389/fpls.2022.975917

**Published:** 2022-12-13

**Authors:** Fabrizio Salonia, Angelo Ciacciulli, Helena Domenica Pappalardo, Lara Poles, Massimo Pindo, Simone Larger, Paola Caruso, Marco Caruso, Concetta Licciardello

**Affiliations:** ^1^ Council for Agricultural Research and Economics (CREA) - Research Centre for Olive, Fruit and Citrus Crops, Acireale, Italy; ^2^ Department of Agriculture, Food and Environment (Di3A), University of Catania, Catania, Italy; ^3^ Research and Innovation Centre, Trento with S. Michele all’ Adige, Trento, Italy

**Keywords:** anthocyanins, β-LCY2, genome editing, antioxidant compounds, regeneration, blood oranges, GoldenBraid 3.0

## Abstract

CRISPR/Cas9 genome editing is a modern biotechnological approach used to improve plant varieties, modifying only one or a few traits of a specific variety. However, this technology cannot be easily used to improve fruit quality traits in citrus, due to the lack of knowledge of key genes, long juvenile stage, and the difficulty regenerating whole plants of specific varieties. Here, we introduce a genome editing approach with the aim of producing citrus plantlets whose fruits contain both lycopene and anthocyanins. Our method employs a dual single guide RNA (sgRNA)-directed genome editing approach to knockout the fruit-specific β-cyclase 2 gene, responsible for the conversion of lycopene to beta-carotene. The gene is targeted by two sgRNAs simultaneously to create a large deletion, as well as to induce point mutations in both sgRNA targets. The EHA105 strain of *Agrobacterium tumefaciens* was used to transform five different anthocyanin-pigmented sweet oranges, belonging to the Tarocco and Sanguigno varietal groups, and ‘Carrizo’ citrange, a citrus rootstock as a model for citrus transformation. Among 58 plantlets sequenced in the target region, 86% of them were successfully edited. The most frequent mutations were deletions (from -1 to -74 nucleotides) and insertions (+1 nucleotide). Moreover, a novel event was identified in six plantlets, consisting of the inversion of the region between the two sgRNAs. For 20 plantlets in which a single mutation occurred, we excluded chimeric events. Plantlets did not show an altered phenotype in vegetative tissues. To the best of our knowledge, this work represents the first example of the use of a genome editing approach to potentially improve qualitative traits of citrus fruit.

## Introduction

Citrus represents a group of species that is highly appreciated by consumers. They have high levels of bioactive compounds (Duarte et al., 2016), such as anthocyanins and carotenoids (e.g., lycopene), which are main sources of antioxidants (Rao and Agarwal, 1999; Krinsky and Johnson, 2005; Smeriglio et al., 2016; Pojer et al., 2020). Anthocyanins reduce inflammation, protect against cardiovascular disease (Mazza, 2007; He and Giusti, 2010), prevent cancer and inhibit its growth, and help fight obesity and type 2 diabetes associated with insulin resistance (Anderson et al., 2004; Cooke et al., 2005; Wang and Stoner, 2008). Lycopene and other carotenoids inhibit lipoprotein oxidation, thus reducing the negative effects of cancer and cardiovascular diseases, inflammatory processes, and Parkinson’s disease (Gerster, 1997; Bramley, 2000). Demand for anthocyanin- or lycopene-pigmented citrus fruits has been increasing, and new varieties have recently been released (Barry et al., 2020).

Anthocyanins are water-soluble compounds synthesized in the phenylpropanoid pathway (Winkel-Shirley, 2001). The biosynthetic pathway and regulatory complex have been comprehensively described in plants (Tanaka et al., 2008; Petroni and Tonelli, 2011). In citrus, *Ruby* (a MYB-like member of the MBW complex) is the crucial transcription factor responsible for the control of anthocyanin production (Butelli et al., 2012; Butelli et al., 2017; Butelli et al., 2019). It is switched on mainly under cold conditions (Lo Piero et al., 2005; Crifò et al., 2011; Carmona et al., 2017) and is light-dependent (Huang et al., 2019). The crucial role of *Ruby* has been demonstrated by its overexpression in non-pigmented citrus fruits, which turns such fruits purple (Dutt et al., 2016). Among citrus fruits, the presence of anthocyanins in so-called blood oranges results in a range of colors from red to purple, characterizing the flesh and the rind of varieties such as Moro’ Tarocco, Sanguigno, and Sanguinello (Caruso et al., 2016). The compound may also change the color of other parts of plant tissues, including young leaves, petals, stamens, styles, and stigmas (Fabroni et al., 2016; Catalano et al., 2020).

The carotenoid pathway in plants is also extensively researched. Biosynthetic genes and their expression profiles, as well as corresponding enzymes, have been described (Kato et al., 2004; Rodrigo et al., 2004; Kato et al., 2006; Tanaka et al., 2008; Chen et al., 2010), although the regulatory genes controlling the pathways are largely unknown. Carotenoid biosynthesis starts from the condensation of two geranylgeranyl diphosphates to produce 15-cis-pythoene, and then a series of desaturations induces the production of lycopene. At this crucial point, the pathway splits into two branches catalyzed by the lycopene cyclase family (*LCY*) genes, in particular epsilon- (*ϵ-LCY*) and beta-cyclase (*β-LCY*); they code for the corresponding enzymes lycopene ϵ-cyclase (*LCYϵ*) and lycopene β-cyclase (*LCYβ*). Both of these are responsible for cyclization and thus degradation of lycopene (Sandmann, 2002). LCYϵ forms monocyclic δ-carotene, which is a substrate used by LCYβ to produce α-carotene. LCYβ can also add two β-rings to lycopene, leading to the biosynthesis of β, β-carotenoids. In citrus, the expression of *LCYs* is different during the transition from chloroplasts to chromoplasts, corresponding to the fruit color break stage, characterized by downregulation of *ϵ-LCY* and overexpression of *β-LCY* (Kato et al., 2004; Alquézar et al., 2008a). Two *β-LCY* genes (*β-LCY1* and *β-LCY2*) have been isolated from sweet orange and grapefruit (Alquézar et al., 2009; Mendes et al., 2011). The *β-LCY1* is expressed in green tissues (leaves, roots, petals, and fruit), while the *β-LCY2* is strongly induced in fruit tissues, particularly in flavedo and pulp (Alquézar et al., 2009). Gene expression and transcriptomic studies on lycopene-pigmented mutants compared to non-pigmented fruits have revealed that lycopene accumulation in pulp is attributable to downregulation of *β-LCY2* gene expression (Alquézar et al., 2008b; Alquézar et al., 2009; Mendes et al., 2011; Alquézar et al., 2013; Liu et al., 2016; Jiang et al., 2019; Lana et al., 2020; Promkaew et al., 2020; Tatmala et al., 2020; Zhang H. et al., 2021).

Some sweet orange varieties (Cara Cara, Hong Anliu, Vaniglia sanguigno, Kirkwood Red), a lemon (Pink fleshed), several pummelos (Chandler, Tubtim Siam, Siam Red Ruby, Thong Dee, Pomelit), and grapefruits (Star Ruby, Flame, Ruby Red, Thompson) are characterized by lycopene accumulation in the pulp, and in some cases also in the rind. The intensity of lycopene pigmentation is rather variable in the different varieties.

To date, no reported study has developed citrus varieties with both anthocyanins and lycopene in their pulp, likely because these traits are difficult to combine through traditional breeding approaches (Salonia et al., 2020). In tomato, the crop with the highest content of lycopene, the activation of the anthocyanin biosynthetic pathway has successfully been performed *via* genetic engineering (Butelli et al., 2008) and traditional breeding (Hazra et al., 2018) approaches. In citrus, traditional breeding takes a long time and requires substantial resources to obtain progeny and evaluate their traits. Moreover, the generation of citrus hybrids that accumulate both compounds is not always feasible because some cultivars are sexually incompatible, sterile, polyembrionic (Talon and Gmitter, 2008), or chimeric (Caruso et al., 2020). One way around this may be to use so-called new plant breeding techniques, which are advanced technologies of genetic engineering that can induce DNA modifications that may be indistinguishable from naturally evolved ones. Genome editing *via* clustered regularly interspaced short palindromic repeats (CRISPR)-associated protein 9 (Cas9) represents a promising strategy to induce a target mutation into a gene of interest controlling a specific trait without modifying the rest of the genome.

Genome editing *via* CRISPR/Cas9 has been widely applied to fruit crops, for example, to induce resistance against *Botrytis cinerea* in grape (Wang et al., 2018) and *Plasmopara viticola* in grapevine (Li et al., 2020), to produce apple varieties resistant to fire blight caused by *Erwinia amylovora* (Pompili et al., 2020), and to produce early-flowering genotypes by knockout Terminal Flower 1 in pear (Charrier et al., 2019) and CENTRORADIALIS in kiwifruit (Varkonyi-Gasic et al., 2019). In citrus, CRISPR/Cas9 has been used exclusively to introduce resistance against citrus canker disease in grapefruit (Jia et al., 2016; Jia et al., 2019) and sweet orange (Wang et al., 2019). Several studies have used genome editing to improve fruit quality (Li et al., 2018a; Xing et al., 2020; Li et al., 2022), and some of them have focused on the lycopene accumulation pathway. Li et al., (2018b) promoted the biosynthesis of lycopene, inhibiting the conversion from lycopene into β- and α-carotene in tomato. Similarly, genome editing of β-cyclase was used to develop a β-carotene-enriched banana variety (Kaur et al., 2020). Following these examples and pursuing an approach that has already been used in tomato (Zsögön et al., 2018; Natalini et al., 2021), we targeted *β-LCY2* using a dual sgRNA approach to produce loss-of-function mutants that stimulate lycopene accumulation in anthocyanin-rich sweet oranges.

## Material and methods

### Plant material

Mature fruits of ‘Carrizo’ citrange (*Citrus sinensis* L Osbeck × *Poncirus trifoliata* L. Raf.) and the ‘Valencia’, ‘Doppio sanguigno’, ‘Vaccaro’, ‘Tarocco TDV’, ‘Tarocco Lempso’, ‘Bud Blood’ sweet orange (*C*. *sinensis* L. Osbeck) were collected from December to February (2019-2020) from the CREA citrus germplasm of Palazzelli (Lentini, Siracusa, Italy; 37°20’22” N, 14°53’31’’ E).

### Optimization of regeneration protocol

Polyembrionic seeds of ‘Valencia’, ‘Doppio Sanguigno’ and ‘Tarocco TDV’ were sterilized with 1% hypochlorite solution and washed three times with sterile water. Then they were sown in tubes containing basal Murashige and Skoog (MS) medium (25 g/L sucrose, 4.4 g/L MS basal medium including vitamins, 7 g/L python agar) and incubated at 25°C under dark conditions for 4–5 weeks. Nucellar seedlings of about 10 cm were recovered from each seed and used for regeneration and transformation experiments. Internodal stem segments were cut and used in regeneration tests. Four different MS media with different hormone concentrations, RDM1, RDM2, RSM1, and RMS2 (**Supplementary Table 1**), were tested to evaluate regeneration efficiency measured after 1 month of culture with a 16 h photoperiod at 25 ± 1°C.

### Citrus transformation protocol

*Agrobacterium*-mediated transformation was carried out for all varieties using internodal stem segments from nucellar seedlings as explants, except in the case of ‘Tarocco Lempso’ for which we used embryogenic calli (obtained from unfertilized ovules cultivated in liquid basal MS medium), and ‘Bud Blood’, for which we used cotyledons (started from sterilized seeds). Transformation experiments were performed using a previously reported method (Orbović and Grosser, 2015) with minor modifications. After *Agrobacterium* infection and co-cultivation, explants were cultured in RDM1 regeneration medium (25 g/L sucrose, 4.4 g/L MS basal medium including vitamins, 1 mg/L BAP) with antibiotics (70 mg/L kanamycin and 400 mg/L cefotaxime) at 25 ± 1°C for 2 weeks under dark conditions and then transferred to a 16 h photoperiod. Explants were transferred to fresh medium every 4 weeks to stimulate the production of transgenic shoots. After 8–10 weeks, shoots were separated from explants and cultured in RDM1 medium with antibiotics to enhance the effect of selection medium. *NptII*-kanamycin-resistant ‘Doppio Sanguigno’, ‘Vaccaro’, ‘Tarocco TDV’, ‘Tarocco Lempso’ and ‘Bud Blood’ shoots were cultured in MS basal medium including vitamins for 2–3 weeks to stimulate plant growth and enhance the efficiency of mini grafts on ‘Carrizo’ rootstock. ‘Carrizo’ shoots, used as transformation control, were cultured in MS medium with 0.5 mg/L NAA for 4–5 weeks to induce rooting.

### Design of sgRNAs

The plasmid for genome editing (pDGB3_alpha1) has been constructed using the GoldenBraid 3.0 system (**Supplementary Table 2**) (Vazquez-Vilar et al., 2017). Two sgRNAs were designed with the coding sequence of *β-LCY2* (FJ516404) and chosen among the most suitable according to the parameters indicated in the Benchling (www.benchling.com) and CRISPR-P 2.0 (Lei et al., 2014; Liu et al., 2017) tools (**Supplementary Table 3**). The criteria used for selection of the sgRNAs were the presence of the same sgRNA in both tools, a distance of no more than 300 bp between the sgRNAs, and an on-target and off-target score higher than 50%. In this study, the sgRNAs were spaced 231 bp from each other, and the on-target score was 71% for sgRNA1 and 55% for sgRNA2 in CRISPR-P 2.0 and 69.8% (sgRNA1) and 50% (sgRNA2) in Benchling. The presence of off-target was verified in both tools, and none of the off-target were found with less than two mismatches in ‘seed region’ of the sgRNA. Although the sgRNAs were designed based on sweet orange (the species that will be edited), we confirmed that no mutations occurred in the ‘Carrizo’ during this process; to do this, a *β-LCY2* sequence was blasted against the *P*. *trifoliata* genome using the Phtyozome blast tool (https://phytozome-next.jgi.doe.gov/blast-search).

### Vector construction

**Figure 1:**
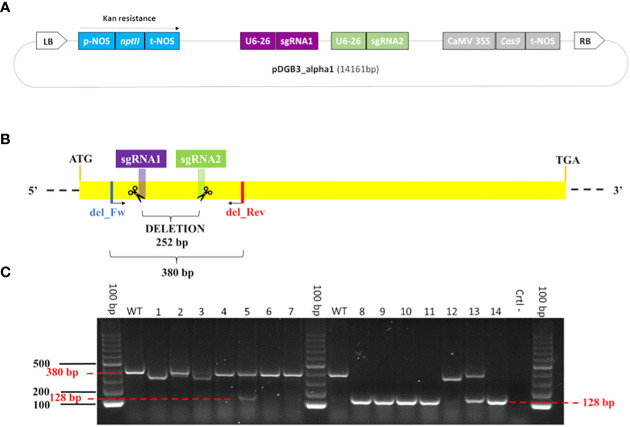
Genome editing construct for the *β-LCY2* gene and validation of edited plantlets. **(A)** Map (not at scale) of the final vector p-DGB3_alpha1 (14.161 bp). From the left border (LB) to the right border (RB), the main portions of the construct are: the *neomycin phosphotransferase II* gene (*nptII*, light blue) with the NOS promoter (p-NOS) and terminator (t-NOS); the two sgRNAs (purple and green) each with the U6-26 promoter; the *Cas9* gene (silver) with the CaMV 35S promoter and NOS terminator. **(B)** Schema of sgRNAs into *β-LCY2*. The size of the deletion between the sgRNAs is 252 bp; the length of the amplicon using the del_Fw and del_Rev primers is 380 bp. **(C)** Gel electrophoresis showing the PCR products of edited plantlets. Samples #4, #6, #7, and #12 show profile I, in which one or both sgRNAs are putatively edited; samples #8, #9, #10, #11, and #14 show profile II, consisting of a large deletion of 252 bp, with an amplicon of 128 bp; samples #5 and #13 show profile III, consisting of two amplicons of 380 bp and 128 bp; samples #1, #2, and #3 show amplicons whose lengths are a little bit different than expected, due to a diverse editing event, and described as profile IV; 100 bp is the ladder; WT is the wild type; Ctrl- is the negative control.

The sgRNAs were domesticated and were used to assemble a CRISPR/Cas9 construct, following the iterative cloning strategy of GoldenBraid 3.0 (Vazquez-Vilar et al., 2017; www.gbcloning.upv.es). Each assembled vector was validated through enzymatic digestion (**Supplementary Figure 1**). The final pDGB3_alpha1 plasmid contained both sgRNAs with a U6 promoter and sgRNA scaffold; the *Cas9* with a 35S promoter and a NOS terminator; and a *nptII* selectable marker gene with a NOS promoter and terminator (**Figure 1A**). The correct assembly of the final vector was checked by Sanger sequencing, using a set of primers that included both sgRNAs (**Supplementary Table 3**). Sanger sequencing was performed using the 3130 Genetic Analyzer (Applied Biosystem) according to a previous study (Arlotta et al., 2020). The vector was used for the transformation of *A*. *tumefaciens* strain EHA105.

### Identification of transformed plants

Resistant shoots were analyzed to detect the presence of *nptII* selectable marker and *Cas9* genes. DNA was extracted using the CTAB protocol with a few modifications (Arlotta et al., 2020). PCR was performed using Taq DNA Polymerase, following the manufacturer’s instructions (VWR, Life Science, Haasrode, Belgium). Amplification conditions were 1 cycle at 95°C for 2 min; 35 cycles at 95°C for 25 s, 57°C for 30 s, and 72°C for 70 s; and a final cycle at 72°C for 5 min. All products were visualized *via* gel electrophoresis (1.5% agarose and 2.5 µL GelRed in 100 mL TAE 1X). Positive ‘Carrizo’ shoots were transferred in jiffy substrate under growth chamber conditions (25 ± 1°C, a 16 h photoperiod, 90% relative humidity), while ‘Doppio Sanguigno’, ‘Vaccaro’, ‘Tarocco TDV’, ‘Tarocco Lempso’, ‘Bud Blood’ shoots were mini grafted on ‘Carrizo’ rootstock (**Supplementary Figure 2**). PCR amplification was repeated with DNA extracted from leaves of mini grafted plantlets after 5 months. The primer sequences are listed in **Supplementary Table 3**.

### Detection of *β-LCY2*-edited plantlets *via* PCR

The genomic DNA of 66 ‘Doppio Sanguigno’, 7 ‘Vaccaro’, 15 ‘Tarocco TDV’, 10 ‘Tarocco Lempso’, 13 ‘Bud Blood’, and 149 ‘Carrizo’ transformed plants was extracted from lateral leaves and apical shoots. The DNA was used as template to amplify a 380 bp fragment using a primers set (**Supplementary Table 3**) designed to amplify the region containing both sgRNAs on *β-LCY2* (**Figure 1B**). The amplification protocol was the same as described above. All PCR products were visualized *via* gel electrophoresis (1.5% agarose and 2.5 µL GelRed (Biotium, Fremont, California, USA) in 100 mL TAE 1X).

### Detection of *β-LCY2* editing events *via* high-throughput sequencing

The *β-LCY2* CRISPR/Cas9-targeted region of 58 transformed citrus lines (36 for ‘Doppio Sanguigno’, 2 for ‘Vaccaro’, 5 for ‘Tarocco TDV’, 2 for ‘Tarocco Lempso’, 5 for ‘Bud Blood’, and 8 for ‘Carrizo’) was screened *via* high-throughput sequencing (HTS). The *β-LCY2* region containing both target sites was amplified using specific primers with overhang Illumina adapters (**Supplementary Table 3**). PCR was performed with 12.5 µL PCRBIO HS Taq Mix Red (PCRBiosystems, London, UK), 0.4 µM each primer, and 25 ng DNA template in a final volume of 25 µL. Amplification conditions were 1 cycle at 95°C for 5 min; 33 cycles at 95°C for 30 s, 55°C for 30 s, and 72°C for 30 s; and a final cycle at 72°C for 5 min.

After purification and quantification, the pooled amplicon libraries were sequenced on an Illumina MiSeq platform (MiSeq Control Software 2.0.5) as reported by Quail et al. (2012). The CRISPResso2 pipeline (https://crispresso.pinellolab.partners.org/submission; Pinello et al., 2016) was used to process the raw paired-end reads (PE300), saved into ‘fastq’ files, to visualize the mutation profiles of the *β-LCY2* target sequence. CRISPResso2 default parameters were used, except for *double guide* mode and *in BATCH* too, and in standalone environment. Moreover, for plantlets coded 11Dk, 15Dk, 32Da, 34Da, 521A, and 7Dk the CRISPResso2 settings were modified using 30% minimum homology for alignment instead of the 60% under default parameter.

For three samples, the CRISPResso2 analysis failed due to the presence of a presumable inversion, even though this event was correctly visualized through basic functions of STAR (Dobin et al., 2013; http://code.google.com/p/rna-star/) and SAMtools. Two primers were designed to confirm the reliability of the inversion (**Supplementary Table 3**) and amplification was performed using Taq DNA polymerase (VWR, Life Science, Haasrode, Belgium), as described above.

## Results

### Optimization of regeneration protocol and identification of the suitable medium for transformation experiments

A minimum of 30 explants of ‘Valencia’, ‘Doppio Sanguigno’, ‘Tarocco TDV’ were separately cultivated in four MS media (RDM1, RDM2, RSM1, and RMS2) (**Supplementary Table 1**). ‘Valencia’ was used as a control because this variety can be efficiently regenerated using available protocols (Boscariol et al., 2003). The regenerant shoots were obtained from indirect organogenesis through callus stage. RDM1 medium showed the highest percentage of explants producing shoots (PEPS) for all varieties (**Supplementary Table 4**); ‘Doppio Sanguigno’ had a PEPS of 90% in both RDM1 and RMS1, higher than that of ‘Valencia’ (80%). RDM1 medium also induced the highest regeneration efficiency in ‘Tarocco TDV’ (**Supplementary Table 4**). Based on these results, we used RDM1 as the most efficient medium for *Agrobacterium*-mediated transformations of all blood orange varieties and ‘Carrizo’ citrange.

### Generation of dual sgRNA construct for the editing of *β-LCY2*

As already mentioned, the genome editing vector was assembled following the interactive cloning strategy of GoldenBraid 3.0 (https://gbcloning.upv.es/), one of the most flexible and feasible approaches for designing such vectors (**Figure 1A**; **Supplementary Table 2**). The assembly of two vectors, each with one of the sgRNAs (Level 1, **Supplementary Figure 1**), required careful evaluation of several colonies; in fact, the integration efficiency of the sgRNAs was low (1 in 30 screened white colonies was positive). The two vectors of Level 2 (**Supplementary Figure 1**), one containing the sgRNA2 cassette and *nptII* (omega_1R) and the other containing sgRNA1 and *Cas9* (omega 2), showed an integration of almost 100%. In our experience, the integration of sgRNAs with U6 promoter and RNA scaffold was more complicated than the assembly of the sgRNAs with *Cas9* and *nptII*. The production of the final vector, consisting of *nptII*, *Cas9*, and both sgRNAs, was validated through PCR using 10 positive colonies, 2 of which were sequenced, producing a 1,220 bp size amplicon (**Supplementary Figure 3**).

### Production of *nptII*- and *Cas9*-positive blood orange varieties

A total of 510 ‘Doppio Sanguigno’, 260 ‘Vaccaro’, 300 ‘Tarocco TDV’, 160 ‘Tarocco Lempso’, 400 ‘Bud Blood’, and 210 ‘Carrizo’ explants were infected with *A*. *tumefaciens*. After about 12 weeks of culturing on selected medium (RDM1 with 70 mg/L kanamycin and 400 mg/L cefotaxime) explants produced kanamycin-resistant shoots as follows: 92 ‘Doppio Sanguigno’, 43 ‘Vaccaro’, 101 ‘Tarocco TDV’, 22 ‘Tarocco Lempso’, 24 ‘Bud Blood’, and 413 ‘Carrizo’ (**Table 1**). The presence of *nptII* and *Cas9* was verified through PCR (**Supplementary Figure 4**). All of the 92 ‘Doppio Sanguigno’ were tested twice, as shoots (coming from the explants) and as plantlets (testing the leaves that resulted from mini grafting). For the other citrus varieties, we were unable to screen all kanamycin-resistant shoots, because some were not recovered due to contamination issues. Therefore, only noncontaminated shoots were mini grafted and screened to confirm to the presence of *nptII* and *Cas9*. Integration of both genes was confirmed in 66 out of 92 ‘Doppio Sanguigno’, 5 out of 7 ‘Vaccaro’, 8 out of 15 ‘Tarocco TDV’, 2 out of 10 ‘Tarocco Lempso’, 9 out of 15 ‘Bud Blood’ and 149 out of 219 ‘Carrizo’, resulting in a transformation efficiency (TE) of 12%, 2%, 2.7%, 1.3%, 2.5%, and 70%, respectively (**Table 1**).

**Table 1:** Transformation efficiency of ‘Doppio sanguigno’, ‘Vaccaro’, ‘Tarocco TDV’, ‘Tarocco Lempso’, ‘Bud Blood’ sweet oranges, and ‘Carrizo’ citrange.

Variety	Infected explants	*nptII* resistant regenerants	Regenerants tested by PCR	*nptII/Cas9* PCR positive	TE*
‘Doppio sanguigno’	510	92	92	66	12%
‘Vaccaro’	260	43	7	5	2%
‘Bud blood’	400	24	13	9	2.5%
‘Tarocco TDV’	300	101	15	8	2.7%
‘Tarocco Lempso’	160	22	10	2	1.3%
‘Carrizo’	210	413	219	149	70%

*The transformation efficiency was calculated dividing the number of regenerants positive for nptII and Cas9 by the number of infected explants.

### Analysis of plantlets with the *β-LCY2* gene

The *nptII*- and *Cas9*-positive plantlets were screened through PCR amplification to detect the large deletion between the two sgRNAs. Overall, we obtained four main profiles (**Figure 1C**; **Supplementary Table 5**): (I) plantlets showing the amplification of one band of 380 bp, presumably having point mutations or short indels within one or both sgRNAs; (II) plantlets in which amplification produced one band of 128 bp, due to the deletion of the region between the sgRNAs; (III) plantlets with two amplicons, whose length were 128 bp and 380 bp; and (IV) other samples displaying amplicons different from 128 bp and 380 bp (**Supplementary Table 6**). Overall, most plantlets (180 out of 239) showed a single amplicon of 380 bp, resulting presumably edited. Moreover, we found 10 plantlets of ‘Doppio Sanguigno’ and 18 of ‘Carrizo’ with the deletion of the region between the two sgRNAs, suggesting a loss-of-function of the *β-LCY2* target gene. Furthermore, 6 ‘Doppio sanguigno’, 1 ‘Tarocco TDV’ and 12 ‘Carrizo’ plantlets showed both amplicons, indicating that they could be heterozygous or chimeric for the editing events. Finally, 12 samples had a profile that could not be classified (see profile IV).

The *β-LCY2* target region was screened from a subset of 50 transformed lines (36 ‘Doppio Sanguigno’, 2 ‘Vaccaro’, 5 ‘Tarocco TDV’, 2 ‘Tarocco Lempso’, 5 ‘Bud Blood’) *via* HTS. One wild-type plant (not subjected to transformation) of each variety was also sequenced and used as a negative (not edited) control. Moreover, we added 8 plantlets of ‘Carrizo’, simply to compare the editing events in the model accession. On average, 24,000 raw sequence reads aligned for anthocyanin-pigmented sweet oranges and 18,000 for ‘Carrizo’ were obtained for each of the analyzed plantlets. This comprehensive coverage supports the accuracy of the editing events produced.

Among the anthocyanin-pigmented sweet oranges, 86% of ‘Doppio Sanguigno’, 100% of ‘Vaccaro’, 100% of ‘Tarocco TDV’, and 80% of ‘Bud Blood’ of successfully transformed plantlets resulted edited and the mutations occurred in the target site of both sgRNAs; The remaining 14% of ‘Doppio sanguigno’ and 20% of ‘Bud Blood’ were not mutated, as well as two plants of ‘Tarocco Lempso’. All of the ‘Carrizo’ plantlets were successfully edited, and mutations were observed in both sgRNAs too (**Supplementary Figure 5A**). The dual sgRNA strategy clearly maximized the modification of citrus plants.

### Detection of point mutations and indels in *β-LCY2*

Most of the analyzed samples showed full knockout, where 100% of the reads were edited. A limited number of mutated plantlets (4 ‘Doppio Sanguigno’, 3 ‘Tarocco TDV’, 1 ‘Bud Blood’, 1 ‘Carrizo’) showed from 11-98% mutated reads, and could be considered heterozygous or more likely chimeric, while the rest were non-mutated reads, identical to the profile of the wild type (**Supplementary Figure 5B**).

Several types of mutations were identified and classified as follows: point insertions (+1 nt), small deletions (-1, -2, -3, -4, -5, -7, -8, -12, -15; -23, -25, -26, -29, -51, -56, -74 nts), and substitution (transition of T into C) (**Supplementary Table 7**). The most frequent type of mutation was an insertion in sgRNA1 and deletion in sgRNA2 (**Supplementary Table 7**; **Supplementary Figure 6**). Therefore, we expect that these indels could produce a frameshift and thus incorrect translation of the protein. **Supplementary Figure 5C** and **Supplementary Table 6** show the mutations in the protein sequence, specifically those of homozygous plantlets with a single editing event (**Supplementary Figure 5B**). Overall, the different editing events caused the introduction of a premature stop codon. A truncated protein is also the result of the deletion of 252 bp, the region between the two sgRNAs (**Supplementary Figure 5C**).

### Inversion, a novel editing event

**Figure 2:**
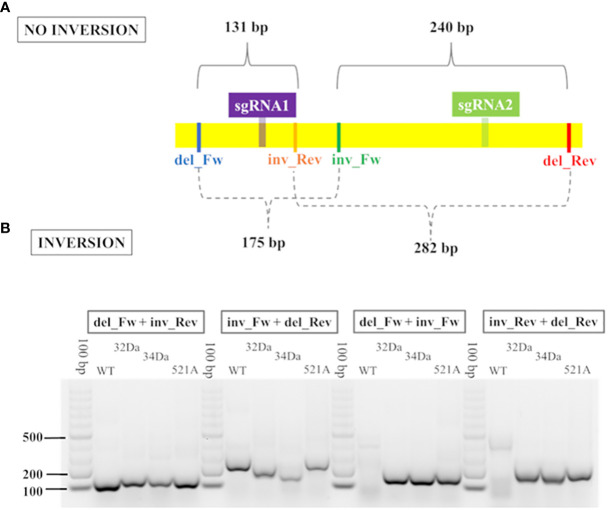
Validation of potential inversion *via* PCR. **(A)** Graphical representation of primers incorporated into the region subjected to potential inversion. Each primer del_Fw and del_Rev was coupled with inv_Fw and inv_Rev, for a total of four different combinations. The length of the amplicons produced by each combination is indicated. **(B)** Gel electrophoresis displays the PCR amplicons obtained through the four primer combinations, used to confirm the potential inversion. WT corresponds to ‘Doppio sanguigno’ wild type, 32Da and 34Da are the codes of ‘Doppio sanguigno’ plantlets, 521A is the code of the ‘Bud Blood’ plantlet.

In addition to point mutations and indels, an unexpected result was observed in two plantlets of ‘Doppio sanguigno’ (32Da, 34Da) and one of ‘Bud Blood’ (521A). For these samples, CRISPResso2 failed to align the reads, classifying the editing event as a putative large insertion, although the amplicon size was of 380 bp. To clarify this unexpected result, we realigned the reads against sweet orange genome 3.0 through SAMtools and STAR and visualized the results using the IGV tool. This is because STAR software, which uses the spliced alignment strategy, can handle large deletion in the amplicon as it does with introns. Surprisingly, it was clear that the region between the sgRNAs was inverted compared to the original sequence. Furthermore, to confirm the *in silico* analysis, we designed two primers within the region subjected to the potential inversion, each one coupled with the primers located upstream of sgRNA1 and downstream from sgRNA2 (**Figure 2A**). The PCR amplification was performed in four different combinations, either considering the absence or the presence of the probable inversion. The wild-type ‘Doppio sanguigno’ exclusively showed the amplification of the noninverted region (73B). In contrast, the three samples with the suspected inversion showed a profile including the wild type and the inversion. Specifically, the presumable wild-type profile of the 521A ‘Bud blood’ could have been due to the very short mutations that occurred in about 35% of the mutated reads (**Supplementary Table 8**); the presence of the inversion was confirmed by the third and fourth primers (**Figure 2B**). A distinct profile occurred in the 32Da and 34Da ‘Doppio sanguigno’ plantlets, in which the presumable wild-type (second primer combination) could be the one typical of plantlets with the length of the amplicon different from 380 bp, as described above (**Supplementary Table 6**).

### Logic procedure behind the identification of additional inversions and large deletions

**Figure 3:**
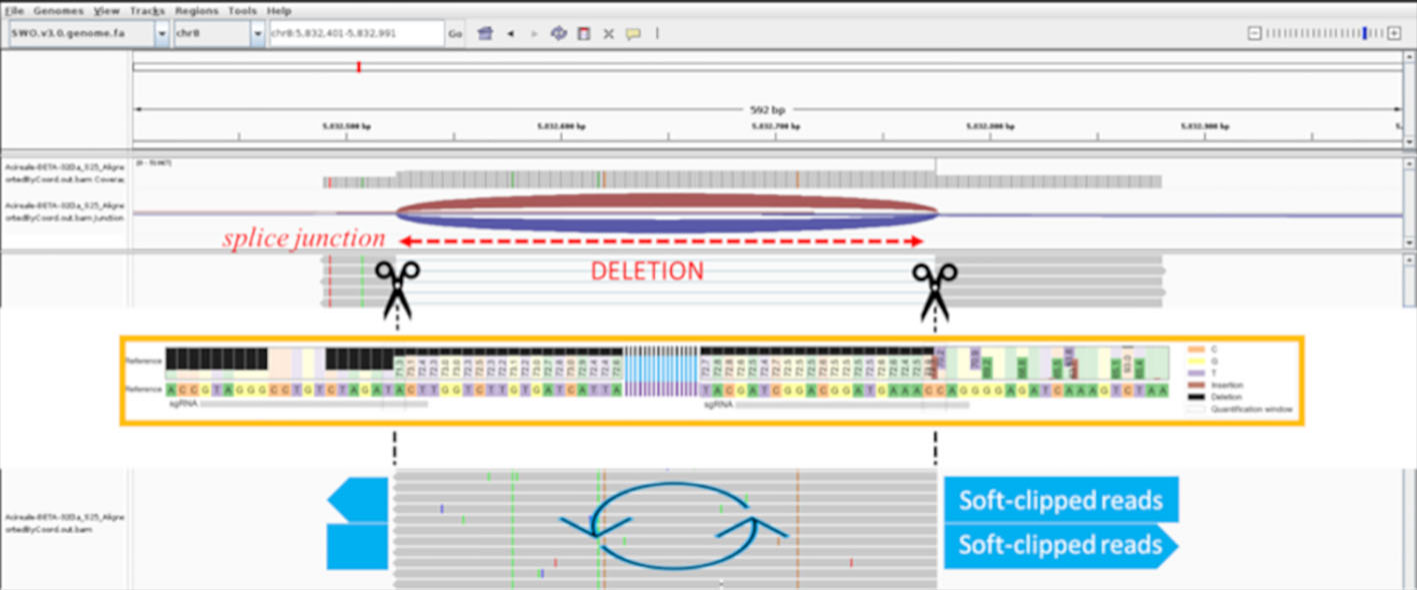
IGV screenshot of the 32Da sample realigned by STAR. The scissors represent the cleavage sites of Cas9, the splice junction shows the deletion (indicated in red), light blue large arrows show the soft-clipped portion of the inverted reads. The orange box refers tois the CRISPResso2 output (not to scale with IGV) of the 32Da with a 30% minimum alignment score; the fragments external to the cleavage sites result in a mutated profile with deletions on the left, and insertions and SNPs on the right (as reported in the legend).

Based on our experience, optimized transformation and regeneration protocols should be followed by a proper bioinformatic analysis to correctly identify induced mutations. Simple detection of editing events is desirable, and the double-guides approach allowed rapid screening through PCR with visualization *via* gel electrophoresis. However, the PCR results contrasted with the first bioinformatic analysis by HTS. The CRISPResso2 tool failed to identify the inversion and the large deletion between the sgRNAs. The insertion visualized by CRISPResso2 contrasted with the band sizes shown in electrophoresis and resulted in the alignment of less than 10 out of 30,000 reads produced by sequencing. We were able to correctly identify the inversion by comparing the PCR/gel results and the CRISPResso2 results using STAR, an additional tool for bioinformatics analysis, which clarified that the large insertion associated with several SNPs (previously visualized by CRISPResso2) corresponded to an inversion. The rationale is that the STAR software, using the spliced alignment strategy, can handle the large deletion in the amplicon as it can with introns. In particular, the amplification of sample 32Da represented the third profile, consisting of a large deletion and a second band similar in size to the one of the wild type. CRISPResso2 analysis displayed an unclear profile for this sample, which looked like a reverse complement of part of the sequence between the two single guides. Approaching the raw reads using STAR produced two alleles, consisting of the deletion represented by splice junctions and the inversion represented as soft-clipped reads between the cutting sites of the guides. In this way, we were able to confirm that the clipped parts were inverted. A schematic of the logic process used to solve the putative inversions and large deletions is shown in **Figure 3**.

CRISPResso2 showed similar results using 30% minimum homology for alignment to the amplicon. Then about 100% of processed reads were correctly aligned for the large deletion; for the inversion, the fragments external to the cutting sites resulted in a mutated profile. PCR confirmed the inversion in all of the suspected samples.

### Sequencing of leaves and apical shoots confirms editing events

Due to the surprisingly high number of homozygous T_0_ citrus plantlets (**Supplementary Figures 5A, B**), we decided to verify if those plants were entirely mutated. To this end, we selected three samples (28Dc and 4DK ‘Doppio sanguigno’, 292A ‘Vaccaro’) previously sequenced, and analyzed them again using about 10 mm apical shoots (a more homogenous sample containing all the meristem layers (Sugawara et al., 2002)) using HTS. Moreover, we added a heterozygous sample (15DK ‘Doppio sanguigno’) as a control, and one of the plantlets showing the inversion (521A ‘Bud Blood’), which gave us the opportunity to verify if the Cas9 continued to cut 7 months after the DNA extraction and amplicon sequencing. The results of the amplicon sequencing confirmed the homozygous profile for the three plantlets (28Dc, 4DK, 292A), as well as the heterozygosity of 15DK, although they indicated a slightly different percentage of editing events. The unexpected complete absences of the wild type *b-LCY2* gene sequence in some of the edited plants could allow to observe the induced phenotype in T_0_. Regarding sample 521A, in the second round of sequencing, the inversion of the region between the two sgRNAs targets was maintained, although the percentage of mutated reads was lower than the previous sequencing. These data are reported in **Supplementary Table 8**.

## Discussion

### Optimization of blood orange regeneration

The application of genome editing technology is possible only if the variety of interest is able to efficiently regenerate after editing. Therefore, much work and resources are needed for the optimization of regeneration and transformation protocols of specific varieties. The unexpectedly high number of escapes may be attributable to a screening performed on acclimated plants, that were not exposed to kanamycin pressure for several weeks; therefore, the resulting positive plants displayed a stable integration of the T-DNA. In citrus, *Agrobacterium*-mediated transformation is the most widely used approach to obtain transgenic plants. In fact, about 90% of transformation experiments are performed using this methodology (Gong and Liu, 2013). However, regeneration of transformed plants represents a bottleneck in citrus, which does not readily regenerate (Cervera et al., 2008). The composition of basal medium and the concentration of hormones positively influence organogenesis response and regeneration efficiency (Cervera et al., 2005; Rodríguez et al., 2008). In our study, the RDM1 medium was the most efficient.

As reviewed in Poles et al. (2020), citrus transformation is accession-specific, easy to perform for ‘Carrizo’ citrange, ‘Duncan’ grapefruit, and ‘Valencia’, ‘Pineapple’ and ‘Jincheng’ sweet oranges. Other species, such as Clementine and sour orange, are considered recalcitrant. Our preliminarily observations suggested that among sweet oranges, ‘Doppio Sanguigno’ was the best genotype to use for transformation. Its transformation and regeneration efficiency were lower than ‘Carrizo’, but still relatively high for editing experiments compared to other varieties tested in the present work. Two previous studies have reported regeneration and transformation protocols for anthocyanin-pigmented citrus varieties. One described transformation of mature tissues of ‘Tarocco’, showing 72.9% and 9.1% regeneration and transformation efficiencies, respectively (Peng et al., 2019). Other study described transformation of young tissues of ‘Maltese half-blood’, in which the regeneration and transformation efficiency was 44.6% and 21.4%, respectively (Jardak et al., 2020). In general, our results are comparable to previous results of other sweet orange genotypes, such as ‘Valencia’ (23.8% TE), Hamlin’ (12.8% TE), ‘Pineapple’ (6.1% TE), and ‘Jincheng’ (4.7% TE) (Cervera et al., 1998; Boscariol et al., 2003; Zou et al., 2013; Orbović and Grosser, 2015). Our work contributes to the optimization of regeneration and transformation protocols for pigmented blood oranges.

### The dual-sgRNA approach, an efficient method to knockout *β-LCY2*

In the last 9 years, since the first study of genome editing technology was published (Gaj et al., 2013), many cloning strategies, adaptable to most applications and plant species, were developed. The generation of genome editing constructs relies on the possibility of using CRISPR/Cas9 plasmids that can be designed using different Cas protein types (Cas9 or Cas12a) or Cas-like and cloning methods (e.g., restriction enzyme ligation, Gateway cloning, Golden Gate assembly). Moreover, different approaches that are able to disrupt gene function could be performed, using one or more sgRNAs (e.g., genome and base editing) to determine base conversions, deletions, insertions, and combination edits introduced into target genomic sites (i.e., prime editing) (Jin et al., 2021). In citrus, the few studies that have applied CRISPR/Cas9 technology used the Golden Gate strategy. This approach has been adopted to introduce resistance to *Xanthomonas citri* (the causal agent of citrus canker disease) to susceptible citrus species (Jia et al., 2016; Jia et al., 2017), to produce a double thorn phenotype in ‘Carrizo’ citrange (Zhang et al., 2021b), and to induce resistance against the herbicide imazapyr (Alquézar et al., 2022).

In our study, we used knockout of the *β-LCY2* gene to combine anthocyanin and lycopene accumulation in the same fruit. The Benchling and CRISPR-P tools were used to design the sgRNAs; therefore, to maximize as much as possible the efficiency of the sgRNAs to select, we chose those that were the same with both tools. Moreover, the same two sgRNAs were sufficiently separated in the target gene and lacked potential off-targets. The dual-sgRNA approach is not always feasible because it is typically only possible when the distance between two sgRNAs is about 300–500 bp. In our case, however, the distance was 231 bp. Generally, the efficiency of mutation increases when more than one sgRNA is designed in a single gene. The nuclease method is the most efficient for causing loss-of-function, leading to the deletion of a large essential part of the gene (Bortesi and Fischer, 2015). The dual-sgRNA approach also has the advantage of easy visual genotyping of mutants based on amplicon length. In our case, 19% of the *nptII*- and *Cas9*-positive plantlets exhibited the deletion between the sgRNAs, producing a truncated protein. Generally, the DNA cleavage efficiency is different among sgRNAs, as are off-target effects. In our study, the mutations in both sgRNAs occurred in 100% of the edited plantlets, leading to efficient inactivation of the gene. Our data indicate that the use of two sgRNAs maximizes the efficiency of gene knockout, similarly to what has been previously reported in pomegranate (Chang et al., 2019) and in citrus using triple guides (Zhang et al., 2020; Zhang F. et al., 2021).

Furthermore, the DNA cleavage by Cas9 at two sites of the gene may induce rearrangements in the gene sequence, as demonstrated by the six independent events of inversion that we observed in the Doppio sanguigno and Bud blood varieties. Inversions mediated by a dual sgRNA CRISPR/Cas9 targeting system have frequently been induced in animal cell lines (Choi and Meyerson, 2014; Essletzbichler et al., 2014; Li et al., 2015), suggesting that this method is a valid way to create targeted inversion mutations and gene deletions. A combined dual sgRNA/Cas9 system was developed for the creation of targeted deletions and gene replacement (Zhao et al., 2016), as well as inversion mutations (Zhang et al., 2016; Blayney et al., 2020) in *Arabidopsis* and in *Oryza* (Liang et al., 2016). In our case, we can speculate that the inversions were a consequence of plasmid design. In fact, the distance between the two sgRNAs in the plasmid and in the *β-LCY2* were similar (260 bp and 252 bp, respectively). A better understanding of this mechanism could lead to a new application of the CRISPR/Cas system. Therefore, we suppose that both sgRNAs, including the scaffold, could be transcribed as unique mRNA. This hypothesis is supported by the fact that the guides were oriented in the same direction. In all of the cases in which the inversion occurred (and probably in other cases), both guides had bonded with the Cas protein to create a unique complex, able to cut portions of gene of around 260 bp. In this way, the complex creates a nick in both sgRNAs, determining three probable options to the DNA repair through the production of small indels, large deletions, and inversions. In particular, the creation of the inversion simultaneously eliminates PAM sites and a portion of the sgRNAs, releasing the cleavage sites.

### Promising fully edited citrus plants

The CRISPR/Cas9 construct was successfully produced, and 49 anthocyanin-pigmented sweet orange edited plantlets were generated. Moreover, 86% of regenerated and transformed plantlets were fully edited; among them, no heterozygous or chimeric events were observed in 43% of transformed ‘Doppio Sanguigno’, 50% of ‘Vaccaro’, 50% of ‘Bud Blood’, and 69% of ‘Carrizo’ plantlets. In these cases, we can hypothesize that the entire plant was mutated, as confirmed by the sequencing of different parts of the plantlets (leaves and apical shoots).

**Figure 4:**
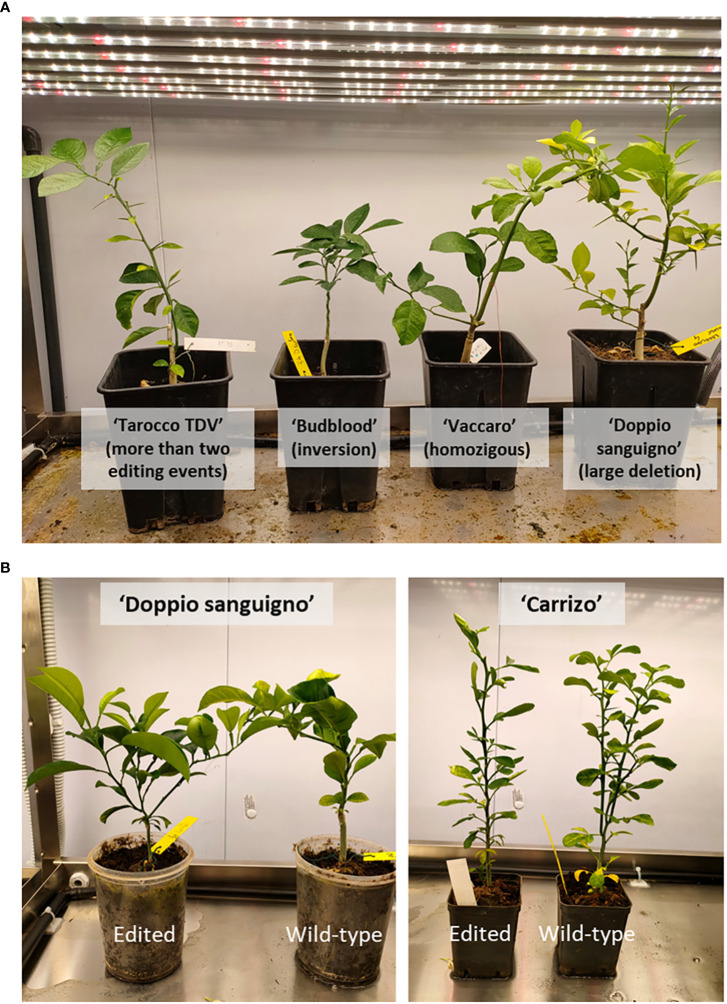
Citrus plantlets edited with the β-LCY2 gene. **(A)** Four editing events in four anthocyanin-rich varieties. Differences in the shapes of plantlets are independent of the mutation type. **(B)** Edited ‘Doppio Sanguigno’ and ‘Carrizo’ plantlets and related wild type. No differences in the phenotype are seen.

Among fully edited plantlets, we place particular attention on those in which a deletion of 252 bp occurred; this produced a specific knockout of *β-LCY2*, leading to the production of a putative truncated protein. A similar result was obtained in plantlets bearing the inversion. Because *β-LCY2* is a fruit-specific chromoplastic gene, we could expect that fruits produced from these plants could be phenotypically mutated. Those plantlets showed a normal phenotype (**Figure 4**), demonstrating that the knockout of *β-LCY2* did not alter the carotenoid metabolism of vegetative tissues.

Theoretically, the Cas9 protein will continue to cut because the recognition site is present in the DNA target (LeBlanc et al., 2018); suggesting that plantlets that are not edited in the first round of validation may be modified after several months. This is what we observed 5 months after the first PCR screening.

## Conclusions

The application of genome editing, mainly if it addressed improving fruit traits of woody plants, is particularly challenging. The data showed in this study add new knowledge for citrus improvement, because genome editing was efficiently adopted to improve qualitative traits of different citrus cultivars. Our strategy generated several edited anthocyanin-pigmented sweet oranges, specifically 38 plantlets of ‘Doppio sanguigno’, 2 of ‘Vaccaro’, 5 of ‘Tarocco TDV’ and 4 of ‘Bud blood’, including non-chimeric genotypes, as supported by HTS analysis. The complete ablation of the functionally active *β-LCY2* gene in T_0_ plants paves the road to the application of this technology to highly heterozygous and vegetatively propagated elite cultivars. For the first time this study reports the transformation of seeds in sweet orange. The transformation protocol for a series of anthocyanin-rich sweet oranges was never tested before. Moreover, the use of the mini grafting in non-sterile environment represents a novelty because it allows acclimation of transformed shoots. The long juvenile stage of citrus plants is a limiting step for the phenotypic evaluation of the edited plants. Grafting the obtained plantlets on early-flowering rootstocks could speed-up fruit production.

## Data availability statement

The data presented in the study are depositated in the NCBI repository, accession number PRJNA853727.

## Author contributions

FS did the constructs and wrote the manuscript; AC supported in all the bioinformatics analysis of editing events, managed the plants and contributed in the writing of the manuscript; HP and LP performed the optimization of transformation and regeneration protocols; MP and SL did the high throughput sequencing; PC reviewed the manuscript; MC grafted the plants and reviewed the manuscript; CL conceived the work and wrote the manuscript. All authors contributed to the article and approved the submitted version.
