# Supplementary material for: A dual sgRNA-directed CRISPR/Cas9 construct for editing the fruit-specific β-cyclase 2 gene in pigmented citrus fruits
Source: Front Plant Sci. 2022 Dec 13;13:975917. doi: 10.3389/fpls.2022.975917 (PMC9792771; doi:10.3389/fpls.2022.975917)
Supplement: Supplementary file 1 [file Presentation_1.pdf]

Supplementary Figure 1. Validation of the assembly of GoldenBraid 3.0 plasmids through enzymatic digestions, as requested for the Level 1 (A) and Level 2 (B). (A) EcoRI was used to confirm the assembly of pDGB3-alpha 1, made up of U6-26 promoter, sgRNA1 and RNA scaffold; HindIII was used to confirm the assembly of pDGB3-alpha 1 including U6-26 promoter, sgRNA2 and RNA scaffold. (B) BamHI was used to confirm the assembly of pDGB3-omega 1, containing the *nptII* selectable marker and the sgRNA2; HindIII was used to confirm the assembly of pDGB3-alpha 2 containing the *Cas9* and the sgRNA1. Gel electrophoresis indicate the profile of *in silico* (on the left) and *on the bench* (on the right) digested plasmids. 'Crtl' is the control represented by the empty vector (alpha1 or alpha2 in (A); omega1 or omega2 in (B)), 'positive' indicates the expected profile derived from *in silico* analyses and red numbers indicate digested plasmids.

Supplementary Figure 2. Workflow of minigrafting procedure on 'Carrizo' rootstock. From left to right, a schematic illustration of the minigrafting; a detail of the minigrafting as it is in the reality; an example of 'Doppio sanguigno' shoot successfully minigrafted.

Supplementary Figure 3. Validation of the final vector assembly through Sanger sequencing. Detail of the plasmid showing the amplicon length of 1,220 bp between the sgRNAs. For each sgRNA, a detail of the chromatogram showing the correct integration obtained through Sanger sequencing is illustrated too.

Supplementary Figure 4. Gel electrophoresis related to the screening by PCR to detect the presence of *nptII* and *Cas9* genes. pDGB3_alpha1 final vector plasmid DNA was used as positive control (Crtl+); 'Doppio sanguigno' wild type DNA was used as negative control (Crtl-). The gel illustrates a subset of samples.

Supplementary Figure 5. High-throughput sequencing data to evaluate *β-LCY2* editing events. **(A)** Edited plantlets of 'Doppio Sanguigno', 'Vaccaro', 'Tarocco TDV', 'Tarocco Lempso', 'Bud blood' sweet oranges, and 'Carrizo' citrange according to mutated reads (100%, from 11 to 98%, no mutations) and the number of editing events (from 0 to more than two; homozygous corresponds to one event). **(B)** Examples of editing events that occurred in each sgRNA as reported in CRISPREsso2 output. The percentage/number of unmodified and modified reads, indicated for each sgRNA, is separated by profile (a, b, c, d). (a) A single event of mutation occurred in 100% of reads (homozygous); (b) two events of mutation in more than 50% and 30% of reads in each sgRNA; (c) more than two editing events; (d) heterozygous plantlets, partially edited; (e) profile typical of non-edited or wild-type plantlets. **(C)** Region from 40 to 180 of 503 amino acids of the translated β-LCY2 protein, including the CRISPR/Cas9 cleavage site. Red shows the different amino acids compared to the wild-type profile. Red asterisks, premature stop codons. Bold, codes of plantlets. Varieties are abbreviated as follows: DS 'Doppio sanguigno', VC 'Vaccaro', BB 'BudBlood', CAR 'Carrizo'. #47 corresponds to the 47 plantlets (16 DS, 1 Tarocco TDV, 30 CAR) that showed the large deletion of 252 bp.

Supplementary Figure 6. Rate of editing (separated in insertions, deletions, and substitutions) occurred in each sgRNA on sweet oranges varieties ('Doppio Sanguigno', 'Vaccaro', 'Tarocco TDV', 'Bud blood') and 'Carrizo' citrange.

Supplementary Table 1. Regeneration media used for the optimization of regeneration protocols.

Supplementary table 2. List of plasmids used to realize the final genome editing vector following the interactive cloning strategy of GoldenBraid 3.0 (https://gbcloning.upv.es/)

Supplementary Table 3. List of primers used in this work. Note indicates the experiments in which primers were used.

Supplementary Table 4. Percentage of explants producing shoots (PEPS) for each genotype on RDM1, RDM2, RSM1 and RSM2 media.

Supplementary Table 5. Validation of edited plantlets performed through PCR to detect the large deletion between both sgRNAs. Four different profiles were classified, depending on the length of the amplicon, and they are separated for each genotype (‘Doppio sanguigno’, ‘Vaccaro’, ‘Tarocco TDV’, ‘Tarocco Lempso’, ‘Bud Blood’ sweet orange varieties, ‘Carrizo’ citrange).

Supplementary Table 6. Type of editing occurred on sgRNA1 and sgRNA 2 on homozygous plants. The plant code, the percentage of mutated reads, the type of mutation (Ins = Insertion, Del = Deletion, Sub = Substitution) and the number of mutated nucleotides (nt) are indicated.

Supplementary Table 7. Number of editing events (insertions, deletions, and substitutions) for each sgRNA observed in ‘Doppio Sanguigno’, ‘Vaccaro’, ‘Bud blood’ sweet oranges and ‘Carrizo’ citrange.

Supplementary Table 8. Results of amplicon sequencing performed on leaves and apical shoots. The plant code, the percentage of total modified (Mod)/unmodified (Unmod) reads, the percentage of mutated reads for each sgRNA, the type of mutation (Ins = Insertion, Del = Deletion, Inv = Inversion; WT = wild type) and the number of nucleotides (nt) for each editing event are reported.
