# Supplementary figures and images for: A dual sgRNA-directed CRISPR/Cas9 construct for editing the fruit-specific β-cyclase 2 gene in pigmented citrus fruits
Source: Front Plant Sci. 2022 Dec 13;13:975917. doi: 10.3389/fpls.2022.975917 (PMC9792771; doi:10.3389/fpls.2022.975917)

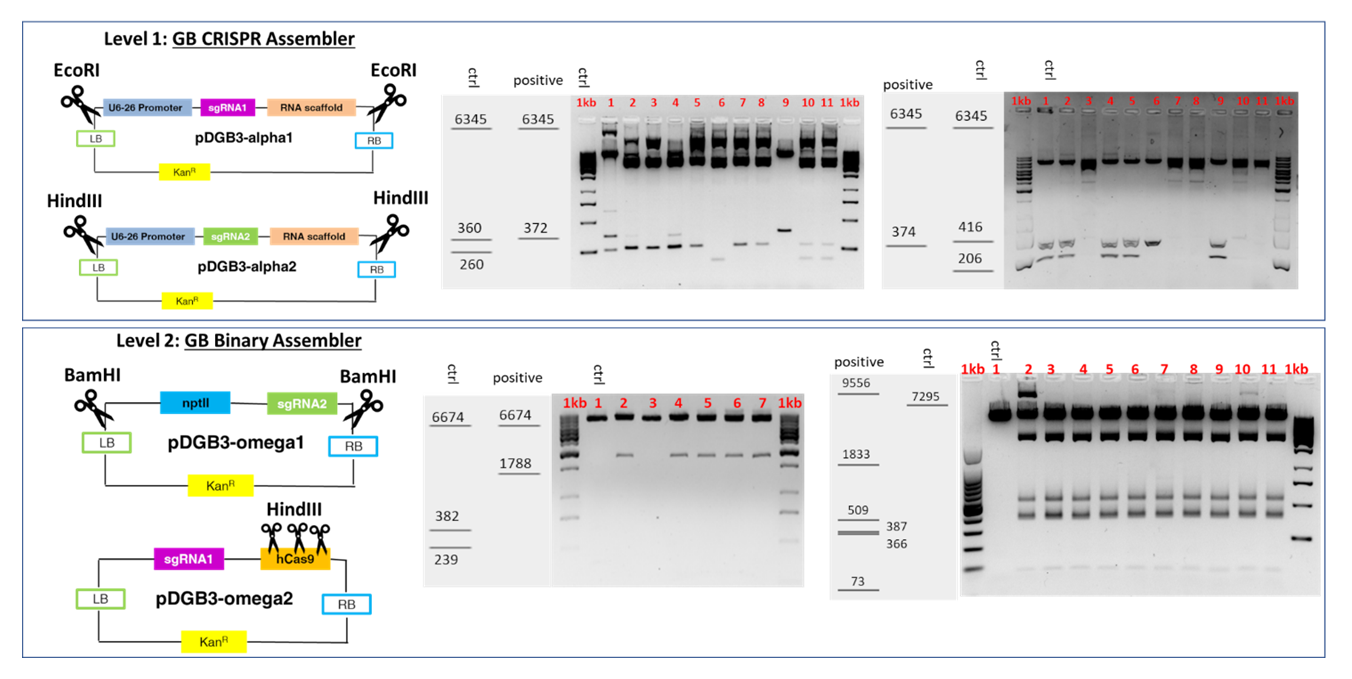

Supplement: Supplementary file 2 [file Image_1.tif]

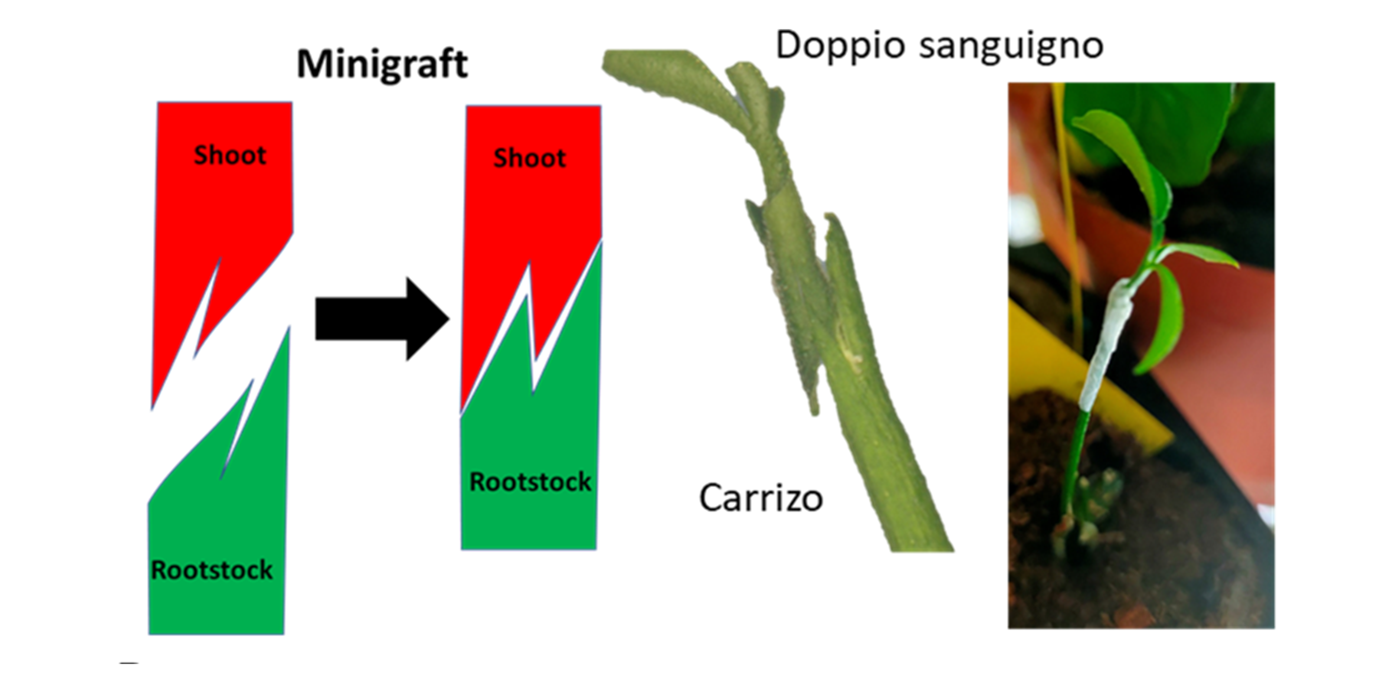

Supplement: Supplementary file 3 [file Image_2.tif]

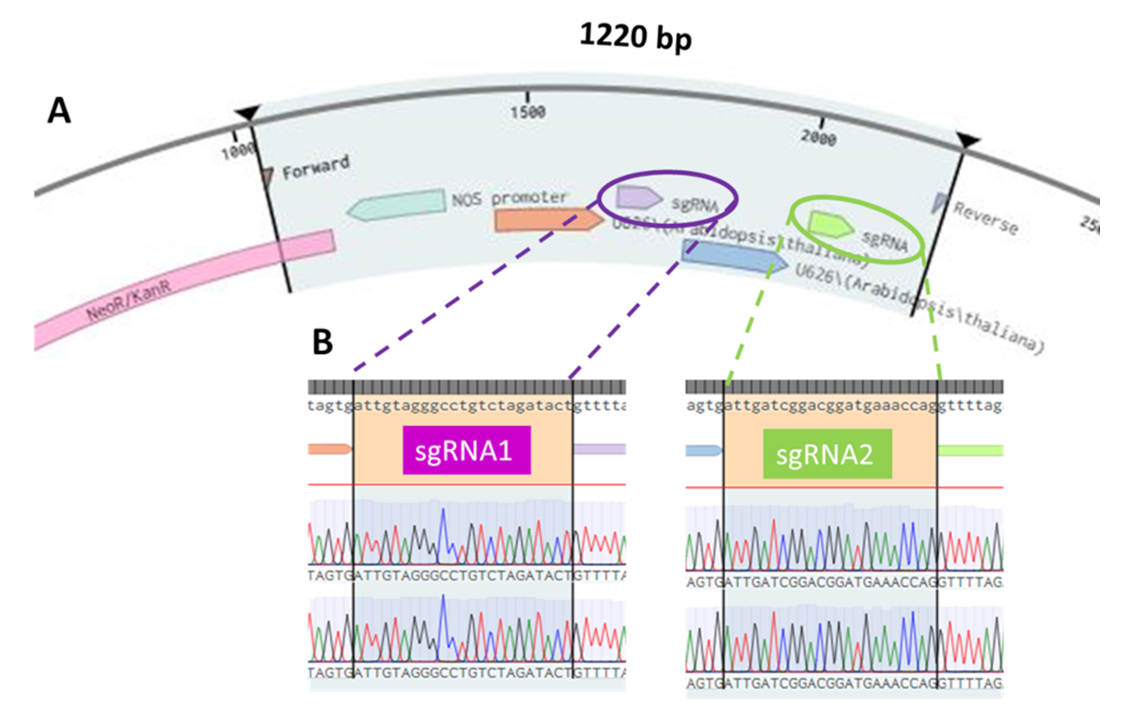

Supplement: Supplementary file 4 [file Image_3.tif]

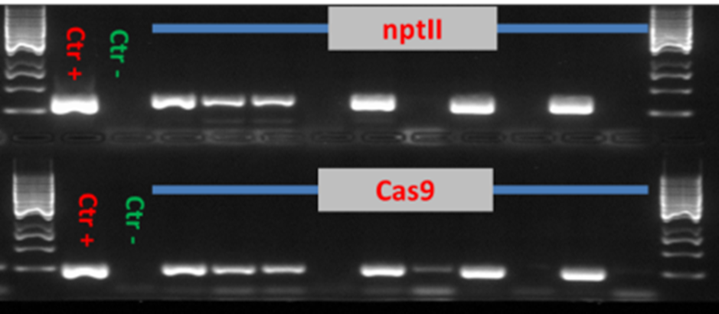

Supplement: Supplementary file 5 [file Image_4.tif]

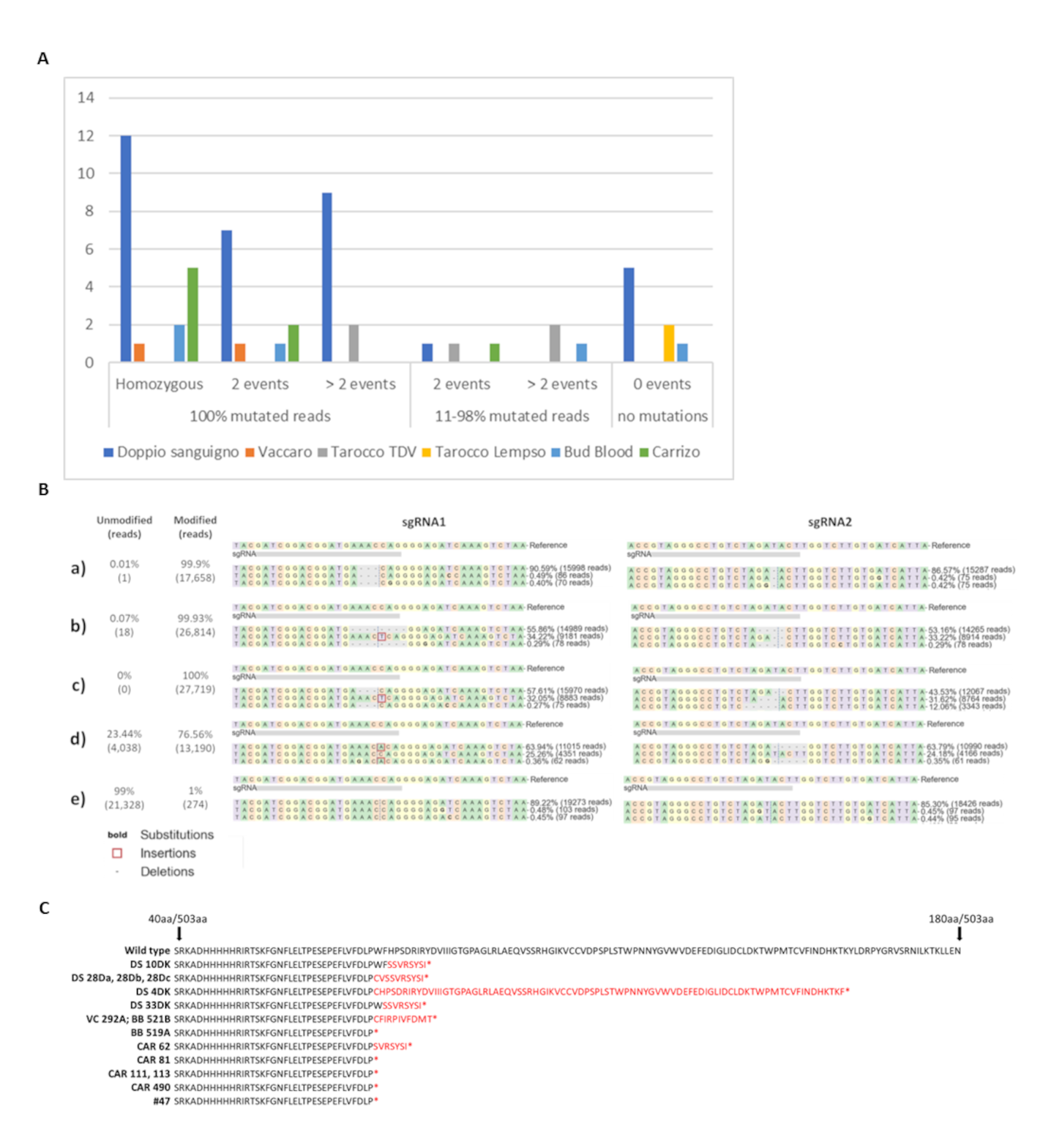

Supplement: Supplementary file 6 [file Image_5.tif]

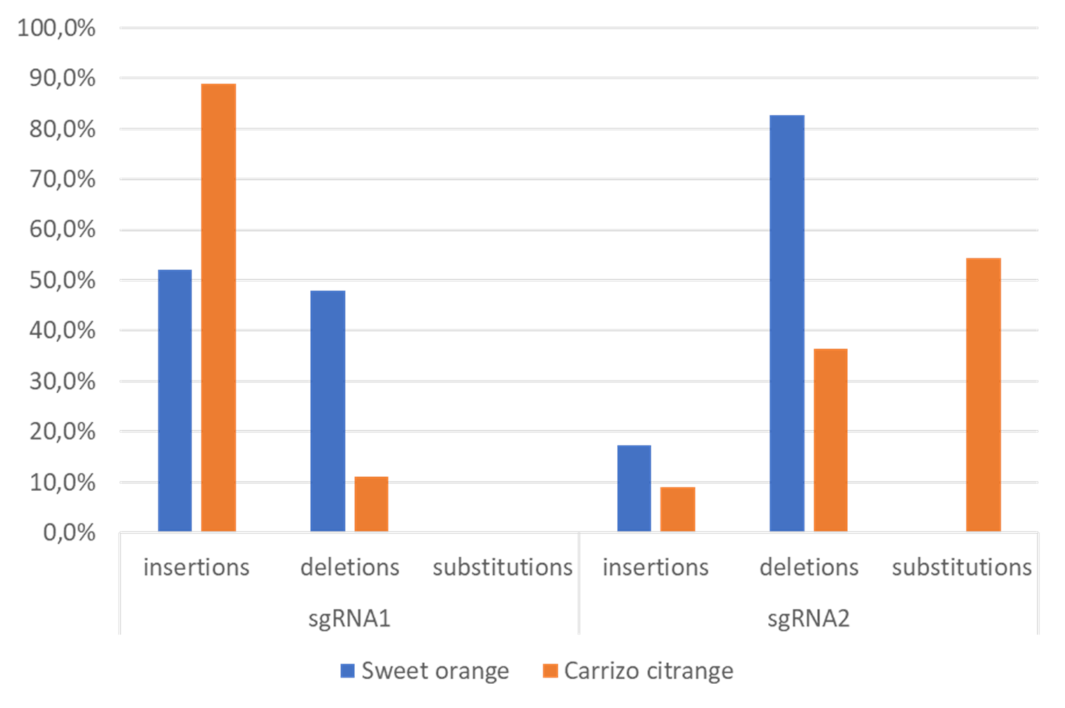

Supplement: Supplementary file 7 [file Image_6.tif]
