# Supplementary material for: A dual sgRNA-directed CRISPR/Cas9 construct for editing the fruit-specific β-cyclase 2 gene in pigmented citrus fruits
Source: Front Plant Sci. 2022 Dec 13;13:975917. doi: 10.3389/fpls.2022.975917 (PMC9792771; doi:10.3389/fpls.2022.975917)
Supplement: Supplementary file 8 [file Table_1.docx]

Supplementary Table 1. Regeneration media used for the optimization of regeneration protocols.

| **Medium** | **BAP concentration** | **NAA concentration** | **References** |
| --- | --- | --- | --- |
| **RDM1** | 1 mg L^-1^ | / | Peña et al., 2001  Dutt and Grosser, 2009  Erpen et al., 2018 |
| **RDM2** | 1 mg L^-1^ | 0.5 mg L^-1^ | Peña et al., 2001  Dutt et al., 2018 |
| **RMS1** | 3 mg L^-1^ | / | Peña et al., 2001  Rodriguez et al., 2008 |
| **RMS2** | 3 mg L^-1^ | 0.5 mg L^-1^ | Peña et al., 2001  Rodriguez et al., 2008  Dutt and Grosser, 2009  Orbović and Grosser, 2015 |
