# Supplementary material for: A dual sgRNA-directed CRISPR/Cas9 construct for editing the fruit-specific β-cyclase 2 gene in pigmented citrus fruits
Source: Front Plant Sci. 2022 Dec 13;13:975917. doi: 10.3389/fpls.2022.975917 (PMC9792771; doi:10.3389/fpls.2022.975917)
Supplement: Supplementary file 9 [file Table_2.docx]

**Supplementary table 2.** List of plasmids used to realize the final genome editing vector following the interactive cloning strategy of GoldenBraid 3.0 (<https://gbcloning.upv.es/>).

| **GB level** | **Accession** | **Name** | **Type** |
| --- | --- | --- | --- |
| Level 0  (GB CRISPR Domesticator) | GB_UC_A2F | sgRNA1 | D Target (B3c-B4-B5c) |
|  | GB_UC_A30 | sgRNA2 | D Target (B3c-B4-B5c) |
| Level 1  (GB CRISPR Assembler) | GB_UA_4966 | pDGB3­_alpha1_sgRNA1 | Transcriptional Units |
|  | GB_UA_4969 | pDGB3_alpha2_sgRNA2 | Transcriptional Units |
| Level 2  (GB Binary Assembler) | GB0226 | pDGB3_alpha1R_Tnos:*nptII*:Pnos | Transcriptional Units |
|  | GB0639 | pDGB3_alpha2_35s:hCas9:tNos | Transcriptional Units |
|  | GB_UA_496D | pDGB3_omega1_*nptII*_sgRNA2 | Module |
|  | GB_UA_496B | pDGB3_omega2_sgRNA1_hCas9 | Module |
|  | GB_UA_496F | pDGB3_alpha1_*nptII*_sgRNA2_sgRNA1_hCas9 | Module |
