# Supplementary material for: A dual sgRNA-directed CRISPR/Cas9 construct for editing the fruit-specific β-cyclase 2 gene in pigmented citrus fruits
Source: Front Plant Sci. 2022 Dec 13;13:975917. doi: 10.3389/fpls.2022.975917 (PMC9792771; doi:10.3389/fpls.2022.975917)
Supplement: Supplementary file 10 [file Table_3.docx]

**Supplementary Table 3**. List of primers used in this work. Note indicates the experiments in which primers were used.

| **Primer name** | **Sequence 5’-3’** | **Annealing temperature** | **Amplicon lenght** | **Note** |
| --- | --- | --- | --- | --- |
| Guide1 | attGATCGGACGGATGAAACCAGgttt | -- | -- | Domestication of single guide. Lowercase indicate GoldenBraid adapters. |
| Guide 2 | attGTAAGGGCCTGTCTAGATACTgttt |  |  |  |
| seq_Fw | CATCAGAGCAGCCGATTGTCT | 58°C | 1220 bp | Validation of the assembly of genome editing vector |
| _seq_Rev | TATCGTCACCTTCTCCGTCG |  |  |  |
| nptII_Fw | CTTCAGCAATATCACGGGTAGC | 57°C | 392 bp | Detection of the presence of *nptII* selectable marker gene in the plant |
| nptII_Rev | GGATCTCCTGTCATCTCACCTT |  |  |  |
| Cas9_Fw | GCATGAACACCAAGTACGATGA | 57°C | 275 bp | Detection of the presence of *Cas9* |
| Cas9_Rev | TATTTCCTGCTCAGACTTTGCG |  |  |  |
| del_Fw | CGGTACCTGAATTCTTAGACTTTG | 55°C | 380 bp | Validation of the presence of large deletion among sgRNAs |
| del_Rev | GTGCCAAACTTTAGCCTTATGAA |  |  |  |
| illumina_Fw | tcgtcggcagcgtcagatgtgtataagagacagCGGTACCTGAATTCTTAGACTTTG | 55°C | 447 bp | Amplificon sequencing of edited plantlets of the *β-LCY2* containing both sgRNAs. Lowercase indicate Illumina adapters. |
| illumina_Rev | gtctcgtgggctcggagatgtgtataagagacagGTGCCAAACTTTAGCCTTATGAA |  |  |  |
| inv_Fw | CCTTCACCTCTTTCTACGTGGCC | -- | -- | Validation of the potential inversion occurred in edited plantlets. Each primer was used in combination with del_Fw and del_Rev. |
| inv_Rev | ACCATGACGCGATGAGACTTG |  |  |  |
