# Supplementary material for: A dual sgRNA-directed CRISPR/Cas9 construct for editing the fruit-specific β-cyclase 2 gene in pigmented citrus fruits
Source: Front Plant Sci. 2022 Dec 13;13:975917. doi: 10.3389/fpls.2022.975917 (PMC9792771; doi:10.3389/fpls.2022.975917)
Supplement: Supplementary file 11 [file Table_4.docx]

**Supplementary Table 4.** Percentage of explants producing shoots (PEPS) for each genotype on RDM1, RDM2, RSM1 and RSM2 media.

| **Genotype** | **PEPS** | | | |
| --- | --- | --- | --- | --- |
|  | **RMS1** | **RMS2** | **RMD1** | **RMD2** |
| ‘Doppio Sanguigno’ | 90.00 | 66.67 | 90.00 | 66.67 |
| ‘Tarocco TDV’ | 61.54 | 53.85 | 84.62 | 14.00 |
| ‘Valencia’ | 70.00 | 50.00 | 80.00 | 43.33 |
