# Supplementary material for: A dual sgRNA-directed CRISPR/Cas9 construct for editing the fruit-specific β-cyclase 2 gene in pigmented citrus fruits
Source: Front Plant Sci. 2022 Dec 13;13:975917. doi: 10.3389/fpls.2022.975917 (PMC9792771; doi:10.3389/fpls.2022.975917)
Supplement: Supplementary file 12 [file Table_5.docx]

**Supplementary Table 5.** Validation of edited plantlets performed through PCR to detect the large deletion between both sgRNAs. Four different profiles were classified, depending on the length of the amplicon, and they are separated for each genotype (‘Doppio sanguigno’, ‘Vaccaro’, ‘Tarocco TDV’, ‘Tarocco Lempso’, ‘Bud Blood’ sweet orange varieties, ‘Carrizo’ citrange).

| **Genotype** | **I** | **II** | **III** | **IV** |
| --- | --- | --- | --- | --- |
|  | **380 bp** | **128 bp** | **380/128 bp** | **≠ 380 / ≠128 bp** |
| ‘Doppio Sanguigno’ | 49 | 10 | 6 | 1 |
| ‘Vaccaro’ | 5 | / | / | / |
| ‘Tarocco TDV’ | 4 | / | 1 | 3 |
| ‘Tarocco Lempso’ | 2 | / | / | / |
| ‘Bud blood’ | 9 | / | / | / |
| ‘Carrizo’ | 111 | 18 | 12 | 8 |
