# Supplementary material for: A dual sgRNA-directed CRISPR/Cas9 construct for editing the fruit-specific β-cyclase 2 gene in pigmented citrus fruits
Source: Front Plant Sci. 2022 Dec 13;13:975917. doi: 10.3389/fpls.2022.975917 (PMC9792771; doi:10.3389/fpls.2022.975917)
Supplement: Supplementary file 13 [file Table_6.docx]

**Supplementary Table 6.** Type of editing occurred on sgRNA1 and sgRNA 2 on homozygous plants. The plant code, the percentage of mutated reads, the type of mutation (Ins = Insertion, Del = Deletion, Sub = Substitution) and the number of mutated nucleotides (nt) are indicated.

| **Plant code** | **sgRNA1** | | | **sgRNA2** | | |
| --- | --- | --- | --- | --- | --- | --- |
|  | **Reads** | **Mutation** | | **Reads** | **Mutation** | |
| **28Da** | 87.73% | Ins | 1 nt | 92.53% | Del | 29nt |
| **28Db** | 87.56% | Ins | 1 nt | 92.94% | Del | 29nt |
| **28Dc** | 87.93% | Ins | 1 nt | 92.91% | Del | 29nt |
| **4DK** | 90.52% | Del | 3 nt | 86.57% | Del | 1 nt |
| **10DK** | 84.40% | Ins | 1 nt | 84.49% | Ins | 1 nt |
| **33DK** | 86.25% | Del | 2 nt | 85.74% | Ins | 1 nt |
| **292A** | 88.95% | Del | 1 nt | 85.69% | Ins | 1 nt |
| **519A** | 89.16% | Ins | 1 nt | 85.25% | Ins | 1 nt |
| **521B** | 89.40% | Del | 1 nt | 85.79% | Ins | 1 nt |
| **62** | 92.37% | Del | 8nt | 86.51% | Del / Sub | 2 nt / T:C |
| **81** | 89.74% | Ins | 1 nt | 89.40% | Del | 7nt |
| **111** | 89.12% | Ins | 1 nt | 84.92% | Sub | T:C |
| **113** | 89.17% | Ins | 1 nt | 86.52% | Sub | T:C |
| **490** | 89.08% | Ins | 1 nt | 90.32% | Del | 13nt |
