# Supplementary material for: A dual sgRNA-directed CRISPR/Cas9 construct for editing the fruit-specific β-cyclase 2 gene in pigmented citrus fruits
Source: Front Plant Sci. 2022 Dec 13;13:975917. doi: 10.3389/fpls.2022.975917 (PMC9792771; doi:10.3389/fpls.2022.975917)
Supplement: Supplementary file 14 [file Table_7.docx]

**Supplementary Table 7**. Number of editing events (insertions, deletions, and substitutions) for each sgRNA observed in ‘Doppio Sanguigno’, ‘Vaccaro’, ‘Bud blood’ sweet oranges and ‘Carrizo’ citrange.

| **Variety** | **Insertions** | | | | **Deletions**  **(bp)** | | | | | | | | | | | | | | | | | **Substitutions**  **(bp)** |
| --- | --- | --- | --- | --- | --- | --- | --- | --- | --- | --- | --- | --- | --- | --- | --- | --- | --- | --- | --- | --- | --- | --- |
|  | **A** | **C** | **G** | **T** | **-1** | **-2** | **-3** | **-4** | **-5** | **-7** | **-8** | **-12** | **-13** | **-15** | **-23** | **-25** | **-26** | **-29** | **-51** | **-56** | **-74** | **T:C** |
| **sgRNA1** | | | | | | | | | | | | | | | | | | | | | | |
| ‘Doppio sanguigno’ | 12 | 1 | 5 | 14 | 2 | 2 | 12 | 1 | 2 | / | 2 | 1 | / | / | / | / | / | / | / | / | / | / |
| ‘Vaccaro’ | / | / | / | 1 | 1 | / | / | / | / | / | / | 1 | / | / | / | / | / | / | / | / | / | / |
| ‘Tarocco TDV’ | 1 | / | / | 1 | 4 | / | 1 | / | / | 3 | / | 1 | / | / | / | / | / | / | / | / | / | / |
| ‘Bud Blood’ | / | / | / | 3 | 2 | / | / | / | / | / | / | / | / | / | / | / | / | / | / | / | / | / |
| ‘Carrizo’ | 1 | 1 | / | 6 | / | / | / | / | / | / | 1 | / | / | / | / | / | / | / | / | / | / | / |
| Total | 14 | 2 | 5 | 25 | 9 | 2 | 13 | 1 | 2 | 3 | 3 | 3 | / | / | / | / | / | / | / | / | / | / |
| **sgRNA2** | | | | | | | | | | | | | | | | | | | | | | |
| ‘Doppio sanguigno’ | / | / | / | 8 | 1 | 12 | 9 | 10 | 15 | 1 | / | / | / | / | 1 | 1 | 1 | 3 | / | / | / | / |
| ‘Vaccaro’ | / | / | / | 2 | / | / | / | / | / | / | / | / | / | / | / | / | / | / | / | 1 | / | / |
| ‘Tarocco TDV’ | / | / | / | 2 | / | 1 | 1 | 1 | / | / | / | / | / | 2 | / | / | / | / | / | 3 | 2 | / |
| ‘Bud Blood’ | / | / | / | 2 | / | / | 1 | / | / | / | / | / | / | / | 1 | / | / | / | / | / | / | / |
| ‘Carrizo’ | / | / | / | 1 | 1 | / | / | / | / | 1 | / | / | 1 | / | / | / | / | / | 1 | / | / | 6 |
| Total | / | / | / | 15 | 2 | 13 | 11 | 11 | 15 | 2 | / | / | 1 | 2 | 2 | / | 1 | 3 | 1 | 4 | 2 | 6 |
