# Supplementary material for: A dual sgRNA-directed CRISPR/Cas9 construct for editing the fruit-specific β-cyclase 2 gene in pigmented citrus fruits
Source: Front Plant Sci. 2022 Dec 13;13:975917. doi: 10.3389/fpls.2022.975917 (PMC9792771; doi:10.3389/fpls.2022.975917)
Supplement: Supplementary file 15 [file Table_8.docx]

**Supplementary Table 8**. Results of amplicon sequencing performed on leaves and apical shoots. The plant code, the percentage of total modified (Mod)/unmodified (Unmod) reads, the percentage of mutated reads for each sgRNA, the type of mutation (Ins = Insertion, Del = Deletion, Inv = Inversion; WT = wild type) and the number of nucleotides (nt) for each editing event are reported.

| **Code** | **Leaves** | | | | | | | | **Apical shoots** | | | | | | | |
| --- | --- | --- | --- | --- | --- | --- | --- | --- | --- | --- | --- | --- | --- | --- | --- | --- |
|  | **Mod.** | **Unmod.** | **sgRNA1** | | | **sgRNA2** | | | **Mod.** | **Unmod.** | **sgRNA1** | | | **sgRNA2** | | |
|  |  |  | **Reads** | **Mutation** | | **Reads** | **Mutation** | |  |  | **Reads** | **Mutation** | | **Reads** | **Mutation** | |
| **28Dc** | 99.99% | 0.01% | 87.93% | Ins | 1 nt | 92.91% | Del | 29nt | 99.99% | 0.01% | 87.76% | Ins | 1nt | 92.46% | Del | 29nt |
| **4DK** | 99.99% | 0.01% | 90.52% | Del | 3 nt | 86.57% | Del | 1nt | 100% | 0% | 91.24% | Del | 3nt | 86.10% | Del | 1nt |
| **292A** | 99.99% | 0.01% | 88.95% | Del | 1nt | 85.69% | Ins | 1nt | 100% | 0% | 88.68% | Del | 1nt | 84.50% | Del | 1nt |
| **15DK** | 87.14% | 12.86% | 19.06% | Del | 1nt | 42.51% | WT | | 91.80% | 8.20% | 29.28% | Del | 1nt | 33.65% | WT | |
|  |  |  | 17.79% | Ins | 1nt | 9.62% | Del | 4nt |  |  | 19.36% | Ins | 1nt | 19.16% | Del | 4nt |
|  |  |  | 12.35% | WT | | 9.55% | Ins | 1nt |  |  | 7.67% | WT | | 6.57% | Ins | 1nt |
|  |  |  | 5.95% | Del | 3nt | 2.93% | Del | 2nt |  |  | 4.60% | Del | 3nt | 3.44% | Del | 2nt |
|  |  |  | 5.85% | Ins | 1nt | 2.26% | Del | 3nt |  |  | 4.19% | Ins | 1nt | 3.42% | Del | 3nt |
|  |  |  | 3.02% | Del | 5nt | 2.02% | Del | 5nt |  |  | 3.76% | Del | 5nt |  | | |
|  |  |  |  | | | | | |  |  | 2.85% | Del | 4nt |  | | |
| **521A** | 100% | 0% | 51.31% | Inv | | 53.14% | Del | 23nt | 99.99% | 0.01% | 54.70% | Del | 6nt | 52.65% | Del | 6nt |
|  |  |  | 35.67% | Ins | 1nt | Del | Del | 3nt |  |  | 34.21% | Inv | | 35.20% | Del | 23nt |
